# Real-world data on breast pathologic complete response and disease-free survival after neoadjuvant chemotherapy for hormone receptor-positive, human epidermal growth factor receptor-2-negative breast cancer: a multicenter, retrospective study in China

**DOI:** 10.1186/s12957-022-02787-9

**Published:** 2022-09-29

**Authors:** Dandan Guan, Qiu Jie, Yihao Wu, Yuhao Xu, Weimin Hong, Xuli Meng

**Affiliations:** 1 General Surgery, Cancer Center, Department of Breast Surgery, Zhejiang Provincial People’s Hospital, Hangzhou Medical College, Shangtang Road No. 158, Hangzhou, 310014 Zhejiang China; 2 Zhejiang Chinese Medical University, Hangzhou, Zhejiang China; 3 Zhejiang University of Technology, Hangzhou, Zhejiang China; 4 Hangzhou Medical College, Hangzhou, Zhejiang China

**Keywords:** Breast pathologic complete response, Hormone receptor-positive/human epidermal growth factor receptor-2-negative breast cancer, Real-world data, Neoadjuvant chemotherapy, Propensity score matching, Nomogram

## Abstract

**Background:**

The data in the real-world setting on breast pathologic complete response (pCR) after neoadjuvant chemotherapy (NAC) for hormone receptor–positive, human epidermal growth factor receptor-2-negative (HR+, HER2−) breast cancer (BC) is limited. The present study aims to screen for some predictors and investigate the prognostic significance of breast pCR after NAC in HR+, HER2− BC in China.

**Methods:**

This was a multicenter, retrospective study. In this study, three hundred eighty-four HR+, HER2− BC patients who received NAC were enrolled between 2010 and 2016 from Shanghai Jiaotong University Breast Cancer Database (SJTU-BCDB). These patients were dichotomized according to the presence of breast pCR after NAC. Logistic analysis was used to screen for predictors associated with breast pCR. Kaplan-Meier (K-M) curve and a propensity score matching (PSM) analysis were performed to compare the disease-free survival (DFS) between the two groups. Cox regression was used to analyze the prognostic significance of breast pCR on DFS in HR+, HER2− BC. A nomogram model was established to predict the probability of DFS at 1, 3, and 5 years after NAC.

**Results:**

Fifty-seven patients (14.8%) achieved breast pCR. Univariate analysis showed that tumor size, estrogen receptor (ER), progesterone receptor (PR), and Ki67 were associated with breast pCR. Further, multivariate analysis showed that tumor size, PR, and Ki67 remained statistically significant. K-M curves showed a statistical difference between the breast pCR and non-pCR groups before PSM (*p* = 0.047), and a more significant difference was shown after PSM (*p* = 0.033). Cox regression after PSM suggested that breast pCR, adjuvant ET, clinical T stage, and Ki67 status were the significant predictive factors for DFS in HR+, HER2− BC patients. The adjusted hazards ratio (aHR) for breast pCR was 0.228 (95% CI, 0.070~0.739; *p* = 0.014), for adjuvant endocrine therapy was 0.217 (95% CI, 0.059~0.801; *p* = 0.022), for Ki67 was 1.027 (95% CI, 1.003~1.052; *p* = 0.027), for cT stages 2 and 3 compared with 1, the values were 1.331 (95% CI, 0.170~10.389), and 4.699 (95% CI, 0.537~41.142), respectively (*p* = 0.043). A nomogram was built based on these significant predictors, providing an integrated probability of DFS at 1, 3, and 5 years. The values of area under the receiver operating characteristic (ROC) curve (AUC) were 0.967, 0.991, and 0.787, at 1 year, 3 years, and 5 years, respectively, demonstrating the ability of the nomogram to predict the DFS.

**Conclusions:**

This real-world study demonstrates that tumor size, PR, and Ki67 were independent predictive factors for breast pCR in HR+, HER2− BC. Breast pCR after NAC was an independent predictor for DFS in HR+, HER2− patients, regardless of a change in nodes. Furthermore, the nomogram built in our study could predict the probability of individualized DFS in HR+, HER2− BC patients.

**Supplementary Information:**

The online version contains supplementary material available at 10.1186/s12957-022-02787-9.

## Background

Neoadjuvant chemotherapy (NAC) is defined as preoperative administration of the systemic cytotoxic treatment and has been the standard care of treatment for locally advanced and inoperable breast cancer (BC) [1]. Nowadays, NAC is being increasingly adopted for patients with operable BC. Some of the previous findings, including those from representative National Surgical Adjuvant Breast and Bowel Project B-18 and B-27, have demonstrated equivalent clinical outcomes with NAC and adjuvant chemotherapy for operable BC [2–4]. NAC is usually administered for the following purposes: downstaging breast tumors and nodes for a less invasive surgery [5–8], assessing clinical response to chemotherapy [9–11], and supporting accelerated approval of novel drugs based on pathologic complete response (pCR) [12, 13].

Patients with hormone receptor-positive (HR+) and human epidermal growth factor receptor-2-negative (HER2−) BC subtype have been less responsive to NAC versus those with other BC subtypes [14–16]. Although surgery is usually preferred over NAC for operable HR+, HER2– patients because of relatively poor clinical response to chemotherapy in this group of patients; however, in patients with operable HR+, HER2− BC who have large tumors or metastatic lymph nodes, NAC is still considered for downstaging the tumors and nodes and thereby providing more favorable surgical options. Thus, the importance of NAC in patients with operable HR+, HER2− BC remains controversial.

Real-world data (RWD) provides insights on patient health status and/or health care delivery in routine clinical practice, including access to treatment, therapeutic efficacy, toxicity, and quality of life, which can help in developing interventions to improve patients’ health care quality, including patients with cancer [17, 18]. A multicenter, retrospective analysis of patients with HR+, HER2− BC who received NAC was conducted, and an RWD was reported on patients’ clinical and pathological characteristics, treatments, and surgical and oncological outcomes in clinical practice. The present study mainly aims to screen for some predictors and prove the prognostic significance of breast pCR after NAC in patients with HR+, HER2− BC by propensity score matching (PSM) approach to offer the integrative and latest data for decision making in the clinic.

## Materials and methods

### Patients and clinicopathological data

**Fig. 1:**
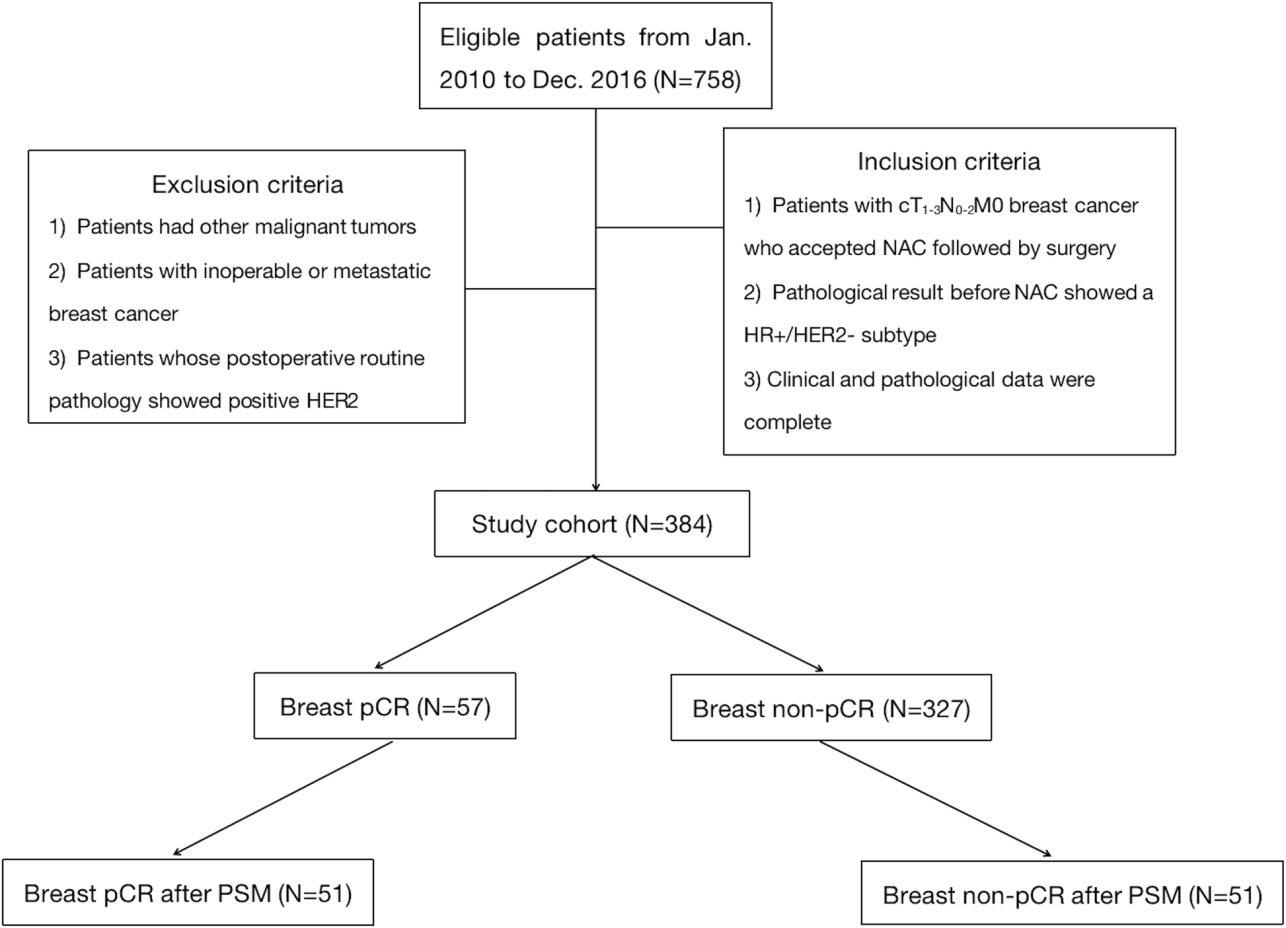
The case selection procedure

This was a noninterventional, multicenter study. Patient baseline clinical characteristics were extracted from the Shanghai Jiaotong University Breast Cancer Database (SJTU-BCDB), which included data from 40 breast cancer centers. Medical records of 758 patients with HR+ BC were reviewed retrospectively from January 2010 to December 2016. Then, patients were selected according to the following inclusion criteria: (1) BC patients in the cT_1-3_N_0-2_M_0_ stage who received NAC followed by surgery, (2) the pathological result prior to NAC showing an HR+, HER2− subtype, and (3) patients were followed for at least 5 years with detailed pathological and clinical data. Patients conforming to the following criteria were excluded: (1) patients with additional cancer, (2) patients who received combined NAC and neoadjuvant endocrine therapy (NET), and (3) patients whose postoperative pathological analysis suggested a HER2+ subtype. Altogether 384 patients with HR+, HER2− BC were finally enrolled. Figure 1 displays the case selection procedure.

### Follow-up data

A total of 20 patients were lost to follow-up during the study, which lasted until December 31, 2021. The clinicopathological features of the patients, such as age, menopausal status, body mass index (BMI), tumor size, histological type, clinical T/N stages, estrogen receptor (ER), progesterone receptor (PR), HER2 status, Ki67 status, treatment patterns (surgery, NAC, radiotherapy, ET, and adjuvant chemotherapy), disease-free survival (DFS), and overall survival (OS) were retrospectively reviewed. The clinical and pathologic stages were defined according to the tumor-node-metastasis (TNM) classification system (8th edition) formulated by the American Joint Committee on Cancer [19]. “T” refers to primary tumor size and the presence/absence of surrounding tissue invasion. “N” refers to the involvement of regional lymph nodes. “M” refers to distant metastasis (DM). DFS is defined as the duration between the primary surgery and the initial recurrence or DM. OS is defined as the duration between primary surgery and all-cause death.

### Immunohistochemical (IHC) and fluorescent in situ hybridization (FISH) analysis

Positive ER or PR stood for the presence of at least 1% of the positive cancer cells among the total number of the cells examined by IHC analysis [20]. In addition, HER2 status was determined through HercepTest as a component of the original histopathological examination. Samples whose IHC scores were 3+ were deemed positive, whereas samples whose IHC scores were 0 or 1+ were considered negative. Inconclusive HER2 results (2+) were subjected to FISH testing for further characterization [21]. According to the ASCO/CAP guidelines, samples with HER2 IHC score 2+ without gene amplification by FISH were considered negative [22, 23].

### Evaluation of pCR and breast pCR

In this study, pCR is defined as no living invasive cancer cells in a nodal basin or primary lesion (ypT_0~Tis_N_0_) [24, 25]. Additionally, breast pCR is defined as the absence of invasive BC in the breast (ypT_0~Tis_) on the final pathological outcomes.

### Statistical analysis

Statistical Package for the Social Sciences (SPSS version 26.0) was utilized for statistical analysis. The differences in clinicopathological features between breast pCR and non-pCR groups were compared by either *χ*^2^ test or *t* test according to feature types. Univariate and multivariate analyses were performed before PSM to identify the predictive factors of breast pCR. In addition, odds ratios (ORs) and the corresponding 95% confidence intervals (CIs) were also determined. Prognostic variables were analyzed by Kaplan-Meier (KM) analysis and log-rank test. Hazard ratios (HRs) along with 95% CIs were determined by Cox regression for ascertaining the influence of breast pCR on DFS in HR+, HER2− BC. The *p* values were two-tailed, and *p* < 0.05 was considered statistically significant.

Based on the significant factors in the Cox regression, a nomogram model was developed to predict the likelihood of DFS. The ability of the model to predict the DFS was measured using receiver operating characteristic (ROC) curve analysis and the area under the ROC curve (AUC). R software was used to generate nomograms, calibration plots, and ROC curves (version 4.1.4).

In order to minimize the selection bias induced by possible confounding factors, a PSM analysis was performed for the comparison between the two groups [26, 27]. In this study, each patient was evaluated through the score determined via possible confounding factors, based on which, the two cohorts were matched. The breast pCR group was compared with the breast non-pCR group in a fair manner, which prevented bias partially. Following PSM variables were selected: age, tumor size, menopausal status, BMI, histological type, ER, PR, HER2, Ki67 status, cT, and cN. In the nearest neighbor matching, a 1:1 ratio in 0.02 standard deviation (SD) of propensity score logit was adopted.

## Results

### Clinical and pathological characteristics of enrolled patients

Overall, 384 patients with cT_1-3_N_0-2_M_0_, HR+, and HER2− BC who received NAC followed by surgery were enrolled for further analysis. A total of 42 patients (10.9%) achieved pCR and 57 (14.8%) patients achieved breast pCR, of which 50 (87.7%) patients were diagnosed as ypT_0_ and 7 (12.3%) patients as ypTis. Out of 251 patients with clinical metastatic lymph nodes, 70 (27.9%) patients were diagnosed as ypN_0_ after surgery following NAC. The patient demographics and baseline characteristics of patients from the 2 cohorts are summarized in Table 1. Statistically significant differences were observed in baseline variables, including BMI, ER, PR, Ki67, adjuvant chemotherapy, ypN stage, LVI, and recurrence between the 2 cohorts of the patients. No statistically significant differences were observed in the baseline variables, including age, menopausal status, tumor size, cT stage, cN stage, histological type, HER2, surgical type, adjuvant ET, adjuvant radiotherapy(RT), and death between the 2 cohorts of patients.

**Table 1:** Baseline characteristics of the HR+/HER2− patients in two cohorts

Variable	Breast pCR (*N*=57)	Breast non-pCR (*N*=327)	*p* value
Age (years)	47.68 ± 9.36	48.87 ± 10.42	
< 50 years	32	174	0.714
≥50 years	25	153	
BMI (kg/m^2^)	23.05 ± 2.98	23.42 ± 2.78	
<18.5	3	6	0.037
18.5~24.9	45	224	
≥25.0	9	97	
Tumor size (cm)	3.46 ± 1.70	3.44 ± 1.39	0.946
Menstrual status			
Premenopause	40	200	0.195
Menopause	17	127	
cT stage			
1	6	19	0.123
2	44	235	
3	7	73	
cN stage			
0	16	117	0.505
1	33	173	
2	8	37	
Histological type			
IDC	53	300	0.809
ILC	3	16	
Others	1	11	
ER status			
<10%	7	16	0.030
≥10%	50	311	
PR status			
<20%	32	121	0.007
≥20%	25	205	
Her2 status (IHC)			
−	13	86	0.150
+	23	160	
++	21	18	
Ki67 status			
<15%	6	88	0.010
≥15%	48	230	
Surgical type			
Mastectomy	52	297	0.922
BCS	5	30	
Adjuvant chemotherapy			
Yes	18	148	0.048
No	39	176	
Adjuvant ET			
Yes	56	301	0.126
No	1	23	
Adjuvant RT			
Yes	39	235	0.524
No	18	89	
ypN stage			
0	40	95	<0.001
1	11	109	
2	5	74	
3	1	48	
LVI			
Yes	3	49	0.045
No	54	278	
Recurrence			
Yes	4	59	0.033
No	51	250	
Death			
Yes	4	30	0.567
No	51	279	

### Treatment characteristics

Treatment characteristics are partly summarized in Table 1. All patients (*N* = 384) received NAC followed by surgery. Of them, 321 (83.6%) patients received NAC containing both anthracycline and taxane, such as the EC-T regimen (four cycles of epirubicin 90 mg/m^2^ + cyclophosphamide 600 mg/m^2^, followed by four cycles of docetaxel 75 mg/m^2^), the TEC regimen (six cycles of epirubicin 75 mg/m^2^ + docetaxel 75 mg/m^2^ + cyclophosphamide 500 mg/m^2^). Of the patients, 267 (69.5%) completed their NAC, and 117 (30.5%) patients had their treatment interrupted. After NAC, 35 (9.1%) patients underwent breast-conserving surgery (BCS), while 349 (90.9%) patients underwent mastectomy. Only 15 (3.9%) patients underwent breast reconstruction. After surgery, adjuvant chemotherapy was administered to 166 (43.2%) patients, most of whom interrupted their NAC. In addition, 274 (71.3%) patients received RT and 357 (93.0%) patients received ET.

### Predictive factors of breast pCR

Univariate analysis showed that the tumor size, ER, PR, and Ki67 status were associated with breast pCR, whereas age, BMI, cT, cN, menstrual status, histological type, and HER2 status were not. Multivariate analysis was conducted to further demonstrate the impact of the related factors for breast pCR, the results of which are shown in Table 2. It was observed that, as continuous variables, a large tumor size (OR = 0.733; 95% CI, 0.588~0.913; *p* = 0.006) and a higher PR (OR = 0.985; 95% CI, 0.976~0.955; *p* = 0.004) were negatively correlated with breast pCR, while a higher Ki67 (OR = 1.019; 95% CI, 1.006~1.032; *p* = 0.004) was positively correlated with breast pCR. No significant correlation was observed between ER and breast pCR in the multivariate analysis.

**Table 2:** Logistic regression analysis of breast pCR-related factors

Variable	Univariate analysis			Multivariate analysis		
	OR	95% CI	*p* value	OR	95% CI	*p* value
Tumor	0.793	0.665~0.966	0.021	0.733	0.588~0.913	0.006
PR status	0.984	0.975~0.993	<0.001	0.985	0.976~0.955	0.004
Ki67 status	1.020	1.008~1.033	0.001	1.019	1.006~1.032	0.004
ER status	0.987	0.978~0.996	0.004			

### Comparison of the prognosis of the patients

**Fig. 2:**
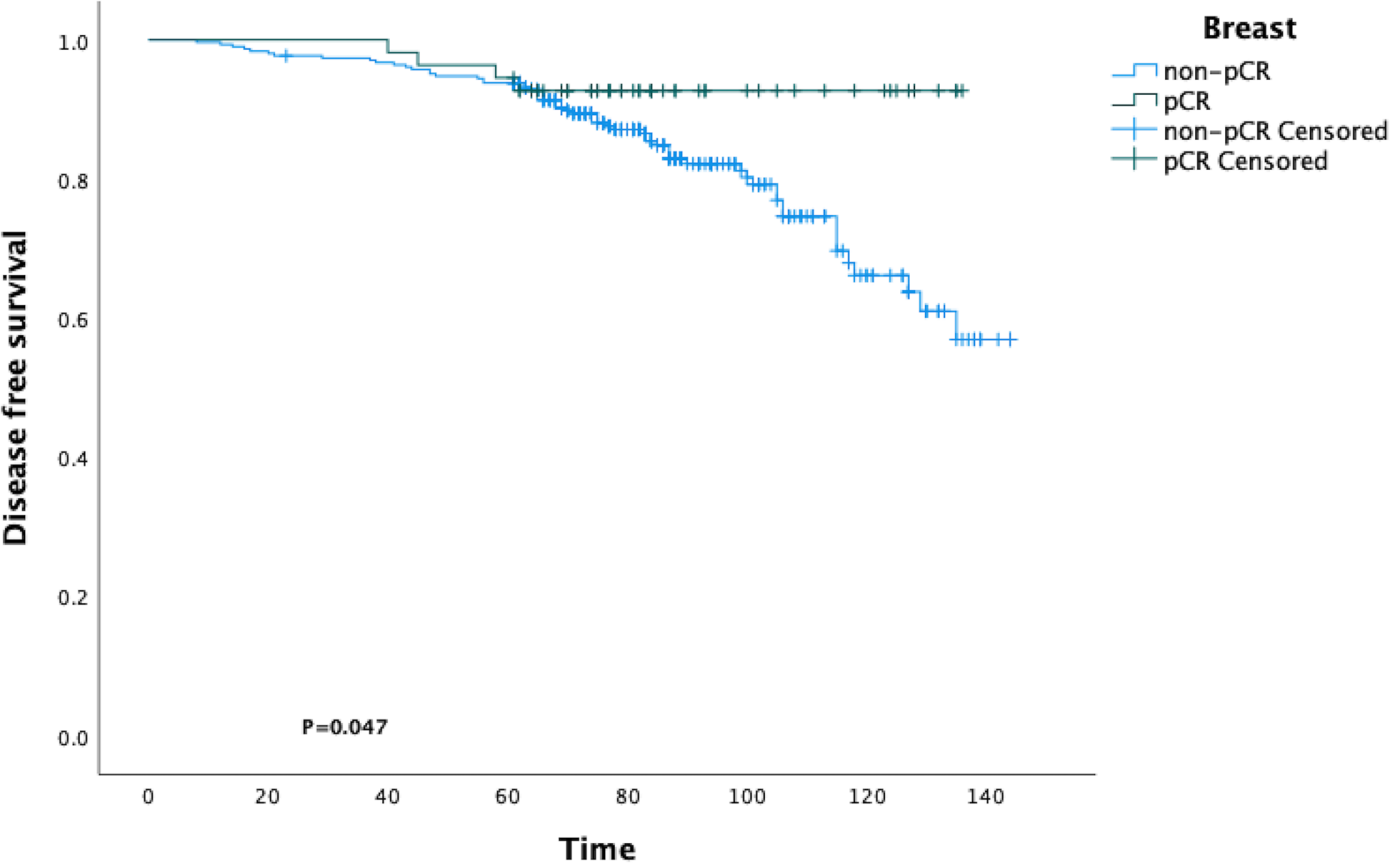
Statistical difference between the breast pCR group and the breast non-pCR group

A total of 20 patients were lost to follow-up over the study period. The median follow-up time was 85.87 ± 25.22 months. During the follow-up, 63 patients experienced relapse, and 34 patients died. The survival curves of DFS were obtained by the KM method. It was observed that there was a statistical difference between the breast pCR group and the breast non-pCR group (*p* = 0.047; Fig. 2). No significant differences were observed in OS between the two groups (*p* = 0.577; Additional file 1: Figure S1). Besides, no significant differences were observed in DFS (*p* = 0.389; Additional file 1: Figure S2A) and OS (*p* = 0.556; Figure S2B) between the pCR group and the non-pCR group.

**Fig. 3:**
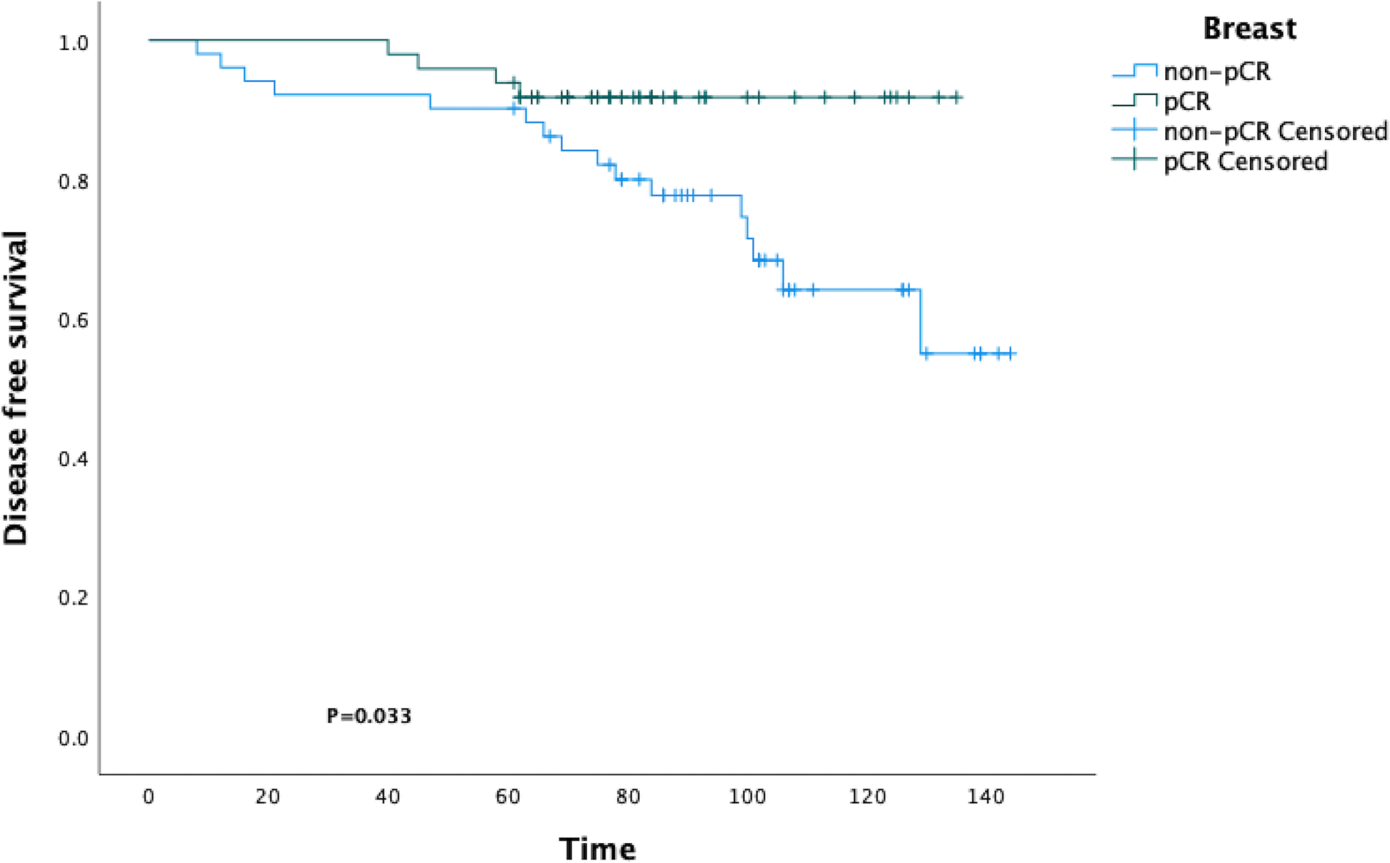
The KM curve and the log-rank test demonstrated statistically significant difference in DFS between the breast pCR group and the breast non-pCR group following PSM

PSM analysis was performed, and a total of 102 cases (51 pairs of matched cases between two cohorts) were enrolled (Table 3), then the DFS was determined for further comparison. The KM curve and the log-rank test demonstrated a statistically significant difference in DFS between the breast pCR group and the breast non-pCR group following PSM (*p* = 0.033; Fig. 3).

**Table 3:** Baseline clinical characteristics and procedure characteristics after PSM

Variable	Breast pCR (*N*=57)	Breast non-pCR (*N*=327)	*p* value
Age (years)	47.90 ± 9.43	49.29 ± 10.60	
< 50 years	37	30	0.664
≥50 years	24	21	
BMI (kg/m^2^)	23.05 ± 2.98	23.42 ± 2.78	
<18.5	3	1	0.180
18.5~24.9	40	33	
≥25.0	8	17	
Tumor size (cm)	3.56 ± 1.71	3.71 ± 1.86	0.642
Menstrual status			
Premenopause	35	35	1.000
Menopause	16	16	
cT stage			
1	4	5	0.543
2	40	41	
3	7	5	
cN stage			
0	13	15	0.521
1	31	24	
2	7	12	
Histological type			
IDC	48	46	-
ILC	3	4	
Others	0	1	
ER status			
<10%	5	3	0.687
≥10%	46	48	
PR status			
<20%	28	34	0.327
≥ 20%	23	17	
Her2 status (IHC)			
−	13	7	0.362
+	21	26	
++	17	18	
Ki67 status			
<15%	6	9	0.549
≥15%	45	42	
Surgical type			
Mastectomy	47	44	0.508
BCS	4	7	
Adjuvant chemotherapy			
Yes	15	23	0.169
No	36	28	
Adjuvant ET			
Yes	50	47	0.250
No	1	4	
Adjuvant RT			
Yes	34	40	0.307
No	17	11	
ypN stage			
0	36	9	<0.001
1	10	16	
2	4	16	
3	1	10	
LVI			
Yes	3	3	1.000
No	48	48	
Recurrence			
Yes	4	16	0.002
No	45	33	
Death			
Yes	4	10	0.146
No	45	39

Cox regression was performed to determine predictive factors for DFS in HR+, HER2− BC. Before PSM, adjuvant ET and ypN stage were found to be predictive factors for DFS. After PSM, it was found that breast pCR, adjuvant ET, cT stage, and Ki67 status were the significant predictive factors for DFS in HR+, HER2− patients. The adjusted HR (aHR) for breast pCR was 0.228 (95% CI, 0.070~0.739; *p* = 0.014), for adjuvant ET was 0.217 (95% CI, 0.059~0.801; *p* = 0.022), for Ki67 was 1.027 (95% CI, 1.003~1.052; *p* = 0.027), and for cT stages 2 and 3 compared with 1, the values were 1.331 (95% CI, 0.170~10.389) and 4.699 (95% CI,0.537~41.142), respectively (*p* = 0.043) Table 4.

**Table 4:** Cox regression for DFS before and after PSM

Variable	Univariate analysis			Variable	Multivariate analysis		
	HR	95% CI	*p* value		HR	95% CI	*p* value
Adjuvant ET	0.226	0.100~0.513	0.000	Adjuvant ET	0.217	0.059~0.801	0.022
ypN stage				cT stage			0.043
0	1[Reference]			1	1[Reference]		
1	0.858	0.396~1.857	0.698	2	1.331	0.170~10.389	0.785
2	2.271	1.115~5.625	0.000	3	4.699	0.537~41.142	0.162
3	4.993	2.436~10.230	0.000	Breast pCR	0.228	0.070~0.739	0.014
				Ki67	1.027	1.033~1.052	0.027

**Fig. 4:**
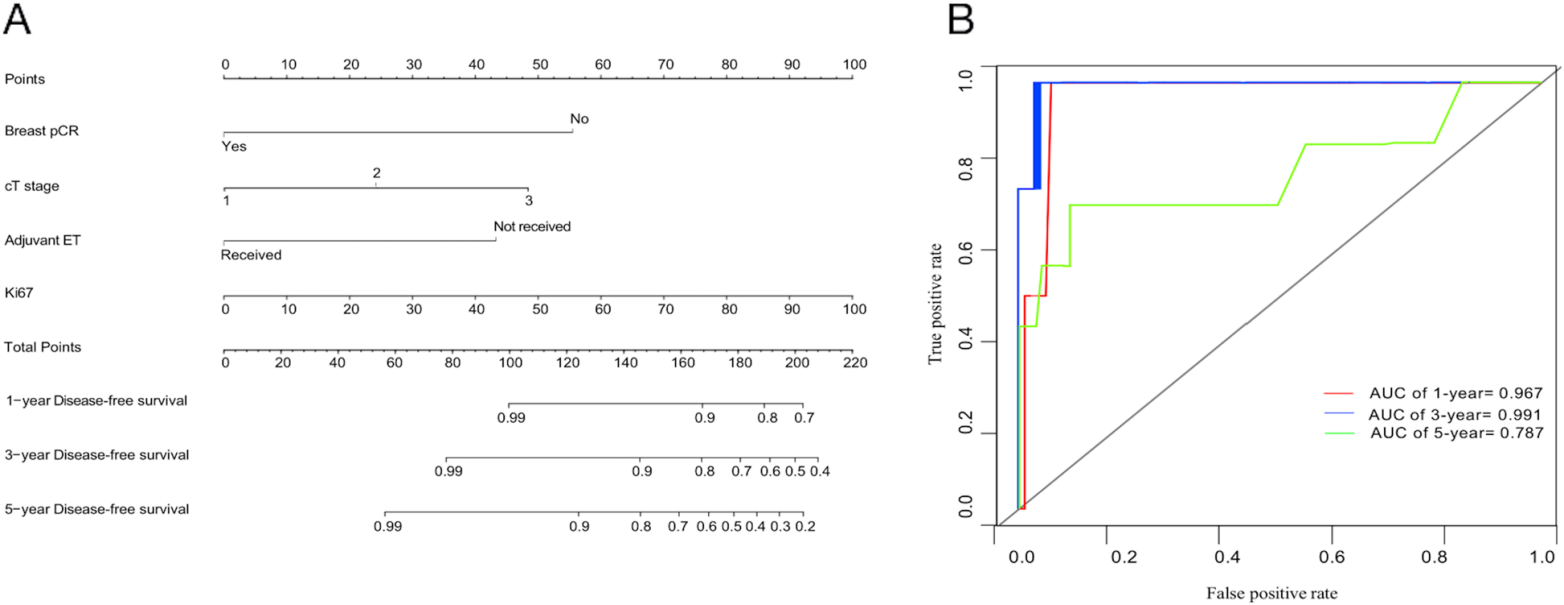
A nomogram model

A nomogram model was developed based on breast pCR, adjuvant ET, cT stage, and Ki67 to assess the probability of DFS at 1, 3, and 5 years after NAC in HR+, HER2− patients (Fig. 4A), providing clinicians with a quantitative method for predicting DFS. Each variable was assigned a point on a scale of 0 to 100 based on the nomogram depicted in this study. Among all variables included, ki67 received a score of 100, followed by breast pCR (yes: score 0; No: score 56), cT stage (T1: score 0; T2: score 24; T3: score 48), and adjuvant ET (received: score 0; not received: score 44). The points for each variable were added to obtain the total points. The total point projected on the bottom scale represents an individual’s likelihood of 1-, 3-, or 5-year DFS after NAC. The model’s ability to predict the DFS was measured using AUC. The values at 1 year, 3 years, and 5 years were 0.967, 0.991, and 0.787, respectively (Fig. 4B), indicating that the model predicting 1- and 3-year DFS was in good agreement with the ideal model.

## Discussion

Currently, NAC is being increasingly used for downstaging tumors and nodes in BC patients. Further, pCR is a well-recognized predictor of survival benefit in triple-negative and HER2-enriched BC subgroups of patients, whereas in the case of luminal BC subtypes, findings are inconsistent [13, 28, 29]. Thus, we reviewed RWD in HR+, HER2− patients attempted to screen the predictors and investigated the prognostic significance of breast pCR after NAC in this subtype.

Findings from the study conducted by Guarneri and colleagues [30] involving 1163 cases demonstrated that patients with HR+ BC who achieved pCR (within nodes and breast) showed better OS (*p* = 0.04) and DFS (*p* = 0.002) than those who did not achieve pCR. In a pooled analysis involving more than 2000 patients with HR+, HER2− BC, a significant difference in event-free survival was observed (HR = 0.49; 95% CI, 0.33~0.71) [13]; nonetheless, such an effect was mainly associated with poor differentiation of tumor achieving a 2-fold increase in pCR rate as compared to the tumors at G1/G2 stages (16.2 vs. 7.5%). However, according to another study involving 417 Japanese patients with HR+, HER2− BC, pCR was not found to be of prognostic value in HR+, HER2− BC [31]. But in BC patients showing transition from cN + stage to pN_0_ following NAC (ypN0), marked increase in OS (*p* = 0.055) and DFS (*p* = 0.004) was observed as compared to patients with pathologic node-positive BC. In the present study, it was found that pCR (within breast and node) had no significant effect on DFS or OS. However, it was observed that patients achieving breast pCR had a better DFS than patients without breast pCR (*p* = 0.047) before PSM, although breast pCR was not associated with a higher OS rate. The prognostic role of breast pCR was further analyzed using PSM to minimize scientific bias due to confounding factors. A significantly positive correlation of DFS with breast pCR was found (*p* = 0.033) after PSM, indicating a better prognosis for the breast pCR group after downstaging tumors by NAC.

This study also showed that tumor size and PR status had a negative correlation with breast pCR, while Ki67 had a positive correlation with breast pCR in HR+, HER2− patients. Some previous reports [32–34] have shown a significant association between ER status, PR status, and pCR. Findings from the study conducted by Lips and colleagues [32] involving 117 patients with HR+, HER2− BC receiving NAC showed that PR-negative cancers were significantly related to the increased pCR rate than PR-positive cancers (7.4 vs. 2.8%; *p* = 0.15), with evidently increased breast pCR/near pCR ratio among PR-negative cases (35.3 vs. 11.7%; *p* < 001). Further, ER status (as a continuous variable) was found to be negatively correlated to the reduction in tumor size by ≥ 50% (OR = 0.99; 95% CI, 0.99~1.00; *p* = 0.027) and pCR (OR = 0.98; 95% CI, 0.97~0.99; *p* < 0.0001). This was in line with the findings of Raphael and colleagues [33], where patients having double HR+ tumors were found to attain pCR than those having one individual HR+ lesion (OR = 0.086; 95% CI, 0.03~0.24; *p* < 0.0001); however, it was not related to long-time survival. Likewise, pooled analysis by van Mackelenbergh and colleagues [34], including 10 neoadjuvant studies carried out by the German Breast Group, demonstrated that PR tumors attained a markedly increased pCR rate than those with double HR+ lesions (11.2 vs. 5.8%, *p* < 0.001) in the HER2− cohort. Cumulatively, these findings suggest that tumors lacking PR might attain pCR among patients with HR+, HER2− BC. In our study, it was observed that a higher PR (as a continuous variable) was associated with a lower breast pCR (OR = 0.985; 95% CI, 0.976~0.955; *p* = 0.004), but lacking PR was not related to a higher breast pCR. Although the univariate analysis suggested a significant correlation between ER and breast pCR (OR = 0.987; 95% CI, 0.978~0.996; *p* = 0.004), no significant correlation was observed using the multivariate analysis.

Previous studies also indicate a potential prognostic value of Ki67 status. In a study by Denkert and colleagues [35], pCR ratio observed in patients with Ki67 < 15% was 3.4%, while patients having Ki67 of 15-35% and > 35% had pCR ratio of 8.2% and 18.5%, respectively, in patients with HR+, HER2− BC (*p* < 0.0005). This was in line with the findings from a previous study on 121 patients with HR+, HER2− BC receiving NAC [36], which demonstrated that Ki67 was related to the pCR ratio within Luminal tumors only. The median Ki67 levels in patients achieving pCR versus patients not achieving pCR were 43% and 29%, respectively (*p* = 0.018). Likewise, in the present study, it was observed that patients a higher Ki67 (as a continuous variable) were associated with a higher rate of breast pCR (OR = 1.019; 95% CI, 1.006~1.032; *p* = 0.004), but no definite threshold has been defined for prediction.

Predictive factors for prognosis were also reported in previous studies. Denkert and colleagues^35^ found in their study that the average OS and DFS was markedly increased among patients with Ki67 < 15% relative to those having Ki67 of 15–35% or > 35% (OS = 8.08 years vs. 7.41 years vs. 6.83 years; *p* = 0.004; DFS = 7.45 years vs. 6.7 years vs. 6.29 years; *p* = 0.04). Additionally, van Mackelenbergh and colleagues [34] reported that PR was the factor that independently predicted DFS, OS, and distant DFS, and the HR values were 1.58 (95% CI, 1.306~1.912; *p* < 0.001), 1.80 (95% CI, 1.406~2.308; *p* < 0.001) and 1.59 (95% CI, 1.299~1.95; *p* < 0.001), respectively. In our study, breast pCR, cT stage, Ki67 status, ypT stage, ypN stage, and adjuvant ET were included as concomitant covariables in cox regression analysis, but tumor size was not, considering that breast pCR was closely related to tumor size. Before PSM, it was found that adjuvant ET and ypN stage were predictive factors for DFS. After PSM, it was observed that breast pCR, adjuvant ET, ki67, and cT stage were statistically significant related factors for DFS in HR+, HER2– patients. The aHR for breast pCR was 0.228 (95% CI, 0.070~0.739; *p* = 0.014), meaning that the breast pCR group had a relatively low recurrence when compared to the breast non-pCR group. In other words, breast pCR after NAC would improve DFS in HR+, HER2– patients.

Based on these significant factors, as concomitant variables, a nomogram was created to estimate the probability of DFS at 1, 3, and 5 years for an individual. It is straightforward to assess the probability of DFS by the nomogram. ROC curves demonstrated the ability of the nomogram to predict the DFS at 1 and 3 years. However, some refinement of the model is still required to improve the predictive ability of DFS at 5 years. Further research focused on validation is in demand to extend the application of the nomogram.

It is sometimes controversial whether one should administer NAC to patients with HR+, HER2− BC, considering the relatively low pCR rate. Based on the results of the present study, it was observed that breast pCR after NAC would bring a DFS benefit in this population, regardless of the change in nodes. Hence, NAC may be worth trying for HR+, HER2− patients with large tumors or metastatic lymph nodes who are already candidates for adjuvant chemotherapy. As the ASCO guidelines recommended, for HR+, HER2− tumors, NAC can be administered instead of adjuvant chemotherapy to any patient in whom the chemotherapy decision can be made without surgical pathology data and/or tumor-specific genomic testing [37]. However, standard clinical and pathological factors, such as tumor size, PR, and Ki67 status, should be taken into consideration together while guiding the treatment decision of NAC. Besides, the nomogram model built in our study is helpful in predicting the probability of DFS for individualized patients.

However, the current study may have several limitations. First, because this was a retrospective study based on the SJTU-BCDB, some potential prognostic parameters, such as multigene signature assessment, and the detailed radiotherapy protocol, were not available in the database. Second, while the current study focused on the prognostic value of breast pCR after NAC in HR+, HER2− patients, more comprehensive studies on the prognostic significance of total pCR or node pCR are needed in the future. Furthermore, the nomogram was developed to predict DFS only for HR+, HER2− BC patients who received NAC, not for other BC patients. Finally, because all of the patients enrolled were all Chinese, the nomogram must be validated in other cohorts. In addition, a PSM analysis was performed in our study to reduce selection bias to some extent through matching, but PSM does not fundamentally solve the statistical problem caused by “selection bias or omission of variables.” As a result, it is suggested that more prospective studies should be conducted and that more prognostic variables should be considered to improve our predictive model.

## Conclusion

Finally, the current study demonstrated that tumor size, PR, and Ki67 status are independent risk factors for breast pCR in HR+, HER2− BC patients. Furthermore, regardless of the change in nodes, breast pCR after NAC will improve DFS. Additionally, as a practical model, the nomogram is useful in predicting the likelihood of individualized DFS after NAC in HR+, HER2− BC patients.

## Supplementary Information

**Additional file 1: Figure S1.** Significant differences were observed in DFS. **Figure S2A.** No significant differences were observed in DFS. Figure S2B no significant differences were observed in OS.

## Data Availability

The raw data supporting the conclusions of this article will be made available by the authors, without undue reservation.

## Abbreviations

PCR: Pathologic complete response
NAC: Neoadjuvant chemotherapy
HR: Hormone receptor
HER2: Human epidermal growth factor receptor-2
BC: Breast cancer
K-M: Kaplan-Meier
PSM: Propensity score matching
DFS: Disease-free survival
ER: Estrogen receptor
PR: Progesterone receptor
HR: Hazard ratio
CI: Confidence interval
ROC: Receiver operating characteristic
AUC: The area under the ROC curve
RWD: Real-world data
SJTU-BCDB: Shanghai Jiaotong University Breast Cancer Database
ET: Endocrine therapy
OS: Overall survival
TNM: Tumor-node-metastasis
DM: Distant metastasis
IHC: Immunohistochemical
FISH: Fluorescent in situ hybridization
ORs: Odds ratios
CIs: Confidence intervals
SD: Standard deviation
BCS: Breast-conserving surgery
