# Supplementary figures and images for: Real-world data on breast pathologic complete response and disease-free survival after neoadjuvant chemotherapy for hormone receptor-positive, human epidermal growth factor receptor-2-negative breast cancer: a multicenter, retrospective study in China
Source: World J Surg Oncol. 2022 Sep 29;20:326. doi: 10.1186/s12957-022-02787-9 (PMC9520808; doi:10.1186/s12957-022-02787-9)

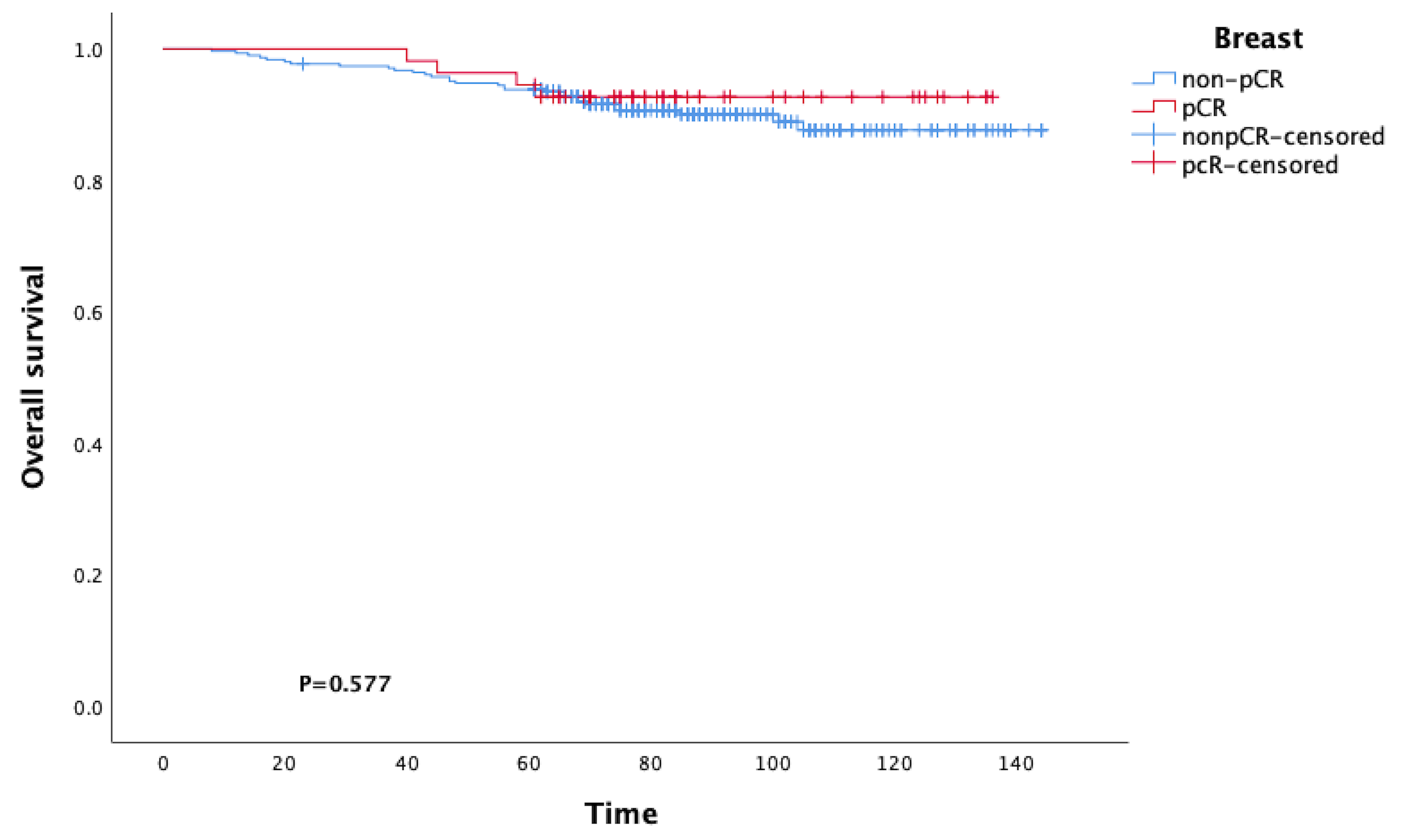

Supplement: Supplementary file 1 — Additional file 1: Figure S1. Significant differences were observed in DFS. Figure S2A. No significant differences were observed in DFS. Figure S2B no significant differences were observed in OS. [file 12957_2022_2787_MOESM1_ESM.zip › Figure S1.jpg]
